# Bioactive baculovirus nanohybrids for stent based rapid vascular re-endothelialization

**DOI:** 10.1038/srep02366

**Published:** 2013-08-06

**Authors:** Arghya Paul, Cynthia B. Elias, Dominique Shum-Tim, Satya Prakash

**Affiliations:** 1 Biomedical Technology and Cell Therapy Research Laboratory, Department of Biomedical Engineering, Faculty of Medicine, McGill University, 3775 University Street, Montreal, Quebec H3A 2B4, Canada; 2 Bulk Manufacturing, Sanofi Pasteur, Connaught Campus, 1755 Steeles Avenue West, Toronto, Ontario, M2R 3T4, Canada; 3 Divisions of Cardiac Surgery and Surgical Research, McGill University Health Center, 687 Pine Avenue West, Suite S8.73, Montreal, Quebec, H3A 1A1, Canada; 4 Current address: Harvard-MIT Division of Health Sciences and Technology, Boston, USA.

## Abstract

Present study, for the first time, reports the development of a nanohybridized baculovirus based stent that can locally promote vascular re-endothelialization by efficient delivery of pro-angiogenic vascular endothelial growth factor (Vegf) genes. *In vitro* data demonstrated rapid expression of functionally active Vegf by the bioactive stent-transduced vascular cells. *In vivo* site-specific transgene expression was observed at the stented regions of balloon-denuded canine femoral artery, which eventually lead to significant endothelial recovery at the injured sites. A significant reduction in neointima formation (2.23 ± 0.56 mm^2^ vs 2.78 ± 0.49 mm^2^ and 3.11 ± 0.23 mm^2^, p < 0.05; n = 8) and percent stenosis was observed in treated stent group compared to negative control and bare metal stent groups. These findings collectively implicate the potential of this newly developed baculovirus based biotherapeutic stent to ameliorate damaged vascular biology and attenuate re-narrowing of stented artery by inhibiting neointima formation.

Percutaneous coronary angioplasty and stenting is one of the most commonly employed interventional procedures for the treatment of coronary artery diseases^1^. A frequent long-term complication of this treatment is the phenomenon of in-stent restenosis (ISR) which occurs at the site of the atherosclerotic lesion, leading to the obstruction of dialated arteries. Designing advanced biotherapeutic material coated intravascular stents for promoting re-endothelialization of damaged stented arteries may offer a promising alternative therapy to the widely used drug eluting (DES) and bare metal stents^2^,^3^,^4^,^5^,^6^,^7^,^8^. DES and metal stents are currently limited by incomplete endothelial recovery due to antiproliferative drugs, inadvertent side effects^9^,^10^,^11^, and increased risk of late-stent thrombosis^12^. Recent studies have demonstrated the importance of such nano-biomaterials to develop new biotherapeutics which have the unique features to restore the natural vascular biology by promoting natural healing in contrary to the DES^13^,^14^,^15^,^16^,^17^,^18^,^19^. Such technologies have the unique potential to promote localized and sustained delivery of biomolecules, such as therapeutic genes, to the damaged arterial wall using the stent surface as the permanent scaffold structure and reservoir for prolonged arterial gene delivery^20^,^21^,^22^. In addition, recombinant baculovirus entrapment to the stent surface allows for increased local concentration of therapeutic agent at the targeted arterial segment without distal spread to non-target tissue, thereby avoiding systemic toxicity and increasing the chance of effective gene transfection to adjacent cells.

The present study, for the first time, introduces a new approach where invertebrate originated, insect cell-specific, recombinant baculovirus nanohybrid materials have been employed for stent based gene delivery to promote vascular re-endothelialization of injured artery (Figure S1). The viral surface has been chemically modified to form cationic nanostructures, similar to our earlier studies^23^,^24^,^25^, for local delivery of human vascular endothelial growth factor-A_165_ (Vegf) therapeutic genes to attenuate ISR^26^. Insect cell host-specific baculovirus (Bac) offers a unique advantage over other gene delivery systems because of its ability to efficiently transduce non-dividing cells, inherent inability to replicate in mammalian cells, low cytotoxicity even at high viral dosage, absence of preexisting antibodies against Bac in animals and also easy production scale up to high viral titre^27^,^28^. On the contrary, the widely experimented mammalian viruses have shown high risks of invoking inflammatory responses and probability for random integration into the host mammalian genome leading to inadequate clinical safety profile^29^. Whereas, as documented in our earlier study, recombinant Bac can be an ideal vector for temporary gene therapy applications such as wound healing and endothelial recovery where the gene expression ceases once its job is done^24^. However, the baculoviruses do not have high transduction efficiency compared to mammalian vectors^30^. Thus genetic and surface modifications of Bac have been under intense research to improve their ability to bind on the mammalian cell surface and improve their lack of specificity, thereby enhancing the transduction efficiency, and overcoming drawbacks of baculoviruses as a gene carrier^31^.

Here, in order to enhance the gene delivery efficiency we have surface-functionalized the Bac with cationic Polyamidoamine dendrimer synthetic nanoparticles (PAMAM) and investigated its effect on transduction efficiency^4^. Furthermore, to protect them from serum inactivation and to achieve a controlled release at the target site, the Bac nanohybrids have been efficiently encapsulated within poly(lactic-co-glycolic acid) (PLGA) microspheres (MS) as optimized in *in vitro* preliminary studies (Figure S2 and Table S1)^32^. The MS were impregnated in fibrin hydrogel and coated using layer-by-layer assembly on prefabricated stent surface^33^, which eventually acts as the scaffold to deliver the protected viral nanohybrids to the target site. We postulated that this new generation of gene eluting stent device can efficiently deliver angiogenic vascular genes to the affected site and induce favorable therapeutic effect in the local vascular biology as illustrated in Figure S1 to promote local wound healing. Accordingly, the study assessed the hypothesis by investigating the stent device efficacy using Vegf carrying recombinant baculovirus (Bac_vegf_) nanohybrids and control Bac carrying no gene (Bac_Null_), followed by evaluating its potential to accelerate re-endothelialization and attenuate in-stent restenosis (ISR) in balloon injured dog femoral artery.

## Results

### Fabrication and characterization of the baculovirus based bioactive stent

For improved gene delivery, as a first step, the anionic Bac particles were surface coated with PAMAM dendrimer to generate the cationic Bac-PAMAM nanocomplexes. The successful formation of Bac-PAMAM nanocomplexes were confirmed by zeta potential analysis of the Bac nanocomplexes as reported in Figure S3a. The data shows that the positively charged PAMAM dendrimers, upon conjugation with the negatively charged baculoviruses, formed nanocomplexes which resulted in an increment of surface charge towards positive. The nanocomplex formation was further confirmed by measuring the particle size of the formed nanocomplexes (Figure S3b). The significantly increased size of the Bac-PAMAM (0.05) particles, where value in brackets represent PAMAM concentration in μmol, compared to the free Bac indicates the efficient formation of Bac-PAMAM nanocomplexes, generated by strong electrostatic interactions between baculovirus and PAMAM dendrimers. The excessively bigger size in Bac-PAMAM (0.1) group is probably because of the formation of larger aggregates and clumps of the formed complexes. Transmission Electron Microcope (TEM) images in Figure S3c demonstrates the efficient binding of the rod like baculovirus with PAMAM dendrimers (0.05) leading to the formation of Bac-PAMAM hybrid complex.

In order to encapsulate the baculoviruses, the method of preparation and energy source required for formation of the primary emulsion were studied extensively and optimized *in vitro*. After optimization (details in supplementary information), we selected double emulsion solvent evaporation method to prepare the virus encapsulated bioactive microparticles^34^. Bac was added to PLGA dissolved in dichloromethane (DCM) and the mixture was either sonicated or homogenized with and without bovine serum albumin (BSA)/glycerol. Hydrophobic (corn oil) solvent was used in the second emulsion in one method (w/o/o) and compared to standard hydrophilic (Polyvinyl alcohol, PVA) solvent method (w/o/w). A significantly higher amount of encapsulation efficiency was noticed in the w/o/o method which confirms that there was less viral loss using oil compared to water in the continuous phase. As evident from Table S1 which summarises the virus encapsulation efficieny and particle diameters from individual preparation methods, we concluded that a w/o/o double emulsion procedure using mechanical homogenizer can efficiently encapsulate the baculoviruses, and this can be further enhanced by supplementing them with BSA/glycerol to protect the viral activity.

**Figure 1:**
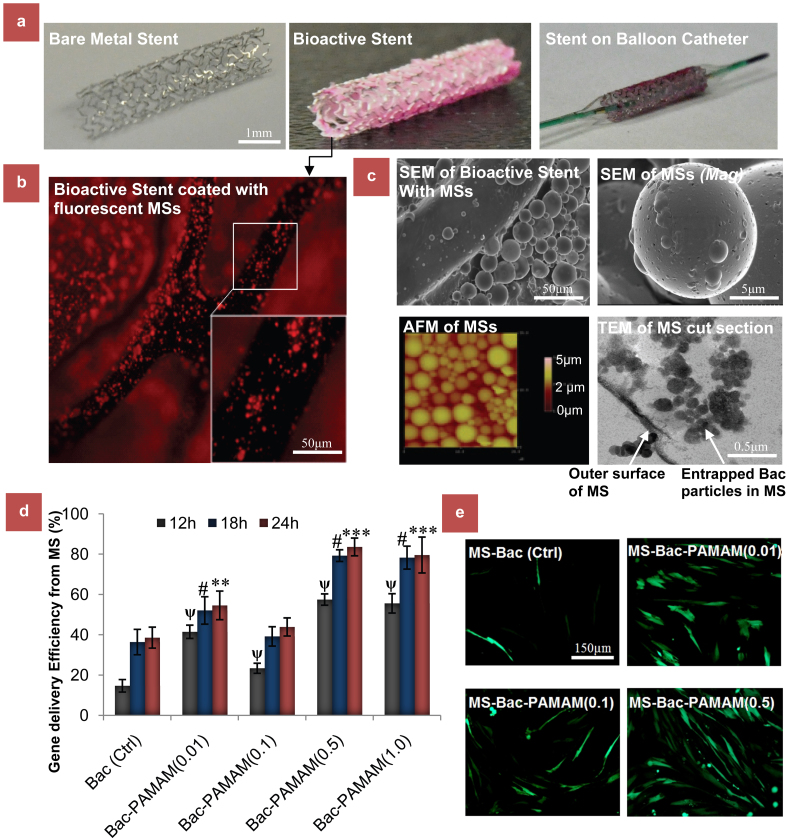
In vitro characterization of bioactive nanohybrid stent platform. (a) Bare metal and bioactive stent with Bac-PAMAM nanocomplex entrapped in MS before and after crimping on balloon catheter. (b) Fluorescence image of the stent showing the fluorescent PLGA MS embedded on the fibrin stent surface. (c) SEM microphotograph of PLGA MS loaded fibrin coated stents, with higher maginification (on right). AFM image demonstrates the surface topography of MSs, encapsulating the nanohybrid baculovirus components. TEM image represents the ultrathin inner cross-sections of the MS encapsulating bioactive viruses. (d*) In vitro* gene delivery efficiency of the bioactive stent in vascular cells. The experimental stents contain Bac_MGFP_ particles, hybridized to 0, 0.01, 0.1, 0.5 and 1.0 μmol PAMAM molecules per 10^8^ baculovirus particles, and incubated in PBS for 24 h. At indicated time points (12 h, 18 h and 24 h) incubated PBS was collected and used for transduction of HASMC using standard procedure mentioned in method section. Fluorescent images on the right represents the GFP expressing transduced HASMCs from different groups, with bac-PAMAM (0.5) group showing highest GFP expression. Data represent mean ± SD (n = 3). ANOVA analysis: * = P < 0.05 and ** = P < 0.01 compared between time (24 h)-matched groups. # = P < 0.05 compared between time (18 h)-matched groups. ψ = P < 0.05 compared between time (12 h)-matched groups.

The prepared polymer coated stents containing the PLGA microspheres (by w/o/o method), mixed with Red Nile dye as tracer, were lyophilized and photographed to confirm that the polymeric stent was able to retain the embedded microspheres after stent expansion. Figure 1a shows a stent before and after coating with MS embedded fibrin layers, while the pink color of the stent confirms retention of the loaded MS on the stent surface post stent expansion using balloon catheter. A fluorescence image of the coated stents in Figure 1b further confirms the above findings, where coating of the microspheres was seen on the stent surface. Scanning Electron Microscope (SEM) and Atomic Force Microscope (AFM) images of the MS on stent surface (Figure 1c** and S4a**) provided further morphological evidences on the surface characteristics of the coated stents demonstrating distributed PLGA MSs embedded on the fibrin multilayered stent struts. Transmission electron microscope (TEM) image of cut section of a virus loaded PLGA microsphere demonstrate the presence of baculovirus inside microspheres which reconfirms the successful microencapsulation.

Preclinical studies have confirmed the safety of using hydrogels, such as fibrin, for coating bomedical devices^35^,^36^. Although it does not posses the ideal mechanical propoerties and biodegradation features needed for proper stent coating, fibrin has been considered as one of the safest biomaterial for tissue engineering and medical appliactions. With that knowledge, here we aimed to develop a biocompatible stent surface by coating the metallic stent with layers of optimized fibrin hydrogel, impregnated with baculovirus loaded biodegradable PLGA MS. The topmost genipin/fibrin layer serves as the barrier to external damages during crimping on the balloon catheter as well as protects the inner layers from premature virus release during its passage through the lumen of the artery at time of implantation at the desired site. The addition of genipin, as a natural cross-linker to fibrin, also works towards reducing the chance of any inflammatory reactions^37^. Through the stent coating method presented here, the inner surface of the stent (i.e., the mandrel contact surface) was not coated with the polymer. Consequently, there was no fibrin coating on the blood contact side of the stent. In this way we tried to reduce the loss of loaded viruses into the blood stream.

To determine the release kinetics of the encapsulated baculoviruses from the stent surface and modulate their release behavior, two different PLGA concentrations (50 and 100 mg/ml of dichloromethne, DCM) were used using w/o/o emulsion method and compared with standard w/o/w method. In both the methods the stent group with MS prepared from lower PLGA concentration showed much faster and higher percentage of viral release with time compared to the higher concentration (Figure S4b). Moreover, w/o/o group showed significantly higher release of active virus at lower PLGA concentration than w/o/w group, 24 h post- incubation in Phosphate Buffered Saline (PBS). A further decrease in PLGA concentration led to non-uniform MS formations and hence we restricted to 50 mg/ml DCM PLGA MS formulation protocol using w/o/o method for further studies.

To investigate whether the released Bac_MGFP_, recombinant baculovirus carrying Monster Green Fluorescent Protein (MGFP) reporter gene, were bioactive and can transduce the human aortic smooth muscle cells (HASMC), the incubation buffers from different release time points were collected, diluted in same proportions and added to HASMC for transduction. After 72 h, the MGFP expressions were quantified in terms of percentage cells transduced. For the study, four different baculovirus formulations using varied concentrations of PAMAM dendrimers were prepared before encapsulating and loading them on the stent surface. The data in Figure 1d–e demonstrates that PAMAM dendrimer functionalization has a positive effect on baculovirus mediated transduction with Bac_MGFP_-PAMAM (0.5) showing consistently higher transduction efficiency than other groups, including free Bac_MGFP_ (control). Further increasing the PAMAM concentration in Bac_MGFP_-PAMAM (1.0) however did not show any significant improvement. Moreover, the percentage of transduction was proportional to hour of incubation buffer. This is because of the higher amount of baculovirus released in the incubation buffer with higher incubation time, although there were no significant differences in transduction efficiencies between 18 h and 24 h in all groups. This indicates that the active viruses were mostly released within 12 h to 18 h from the stent. Furthermore, 72 h cytotoxicity studies on the effect of these different formulations on HASMC show that Bac_MGFP_-PAMAM can be safely used for cell transduction, although Bac_MGFP_-PAMAM (1.0) group with high PAMAM concentration showed significantly high cytotoxicity compared to the control free Bac and other experimental groups (Figure S5).

Importantly, the stent group with microencapsulated Bac-PAMAM also showed significantly higher protection of bioactive baculoviruses against serum inactivation compared to stents with microencapsulated Bac, and non-encapsulated Bac and Bac-PAMAM stents. Our data confirms that even PAMAM coating can also protect the Bac against serum inactivation to some extent, which is further enhanced by encapsulating them inside PLGA microspheres (Figure S6a). Additionally, the preservation of bioactivity of the stent for at least 3 months stored at −80°C, as illustrated in Figure S6b, can be of significant logistic advantages under real life clinical settings as the stored stent can be of immediate off-the-shelf use for any patient undergoing angioplasty and stenting without delay.

### *In vitro* biofunctionality of the developed bioactive stent

**Figure 2:**
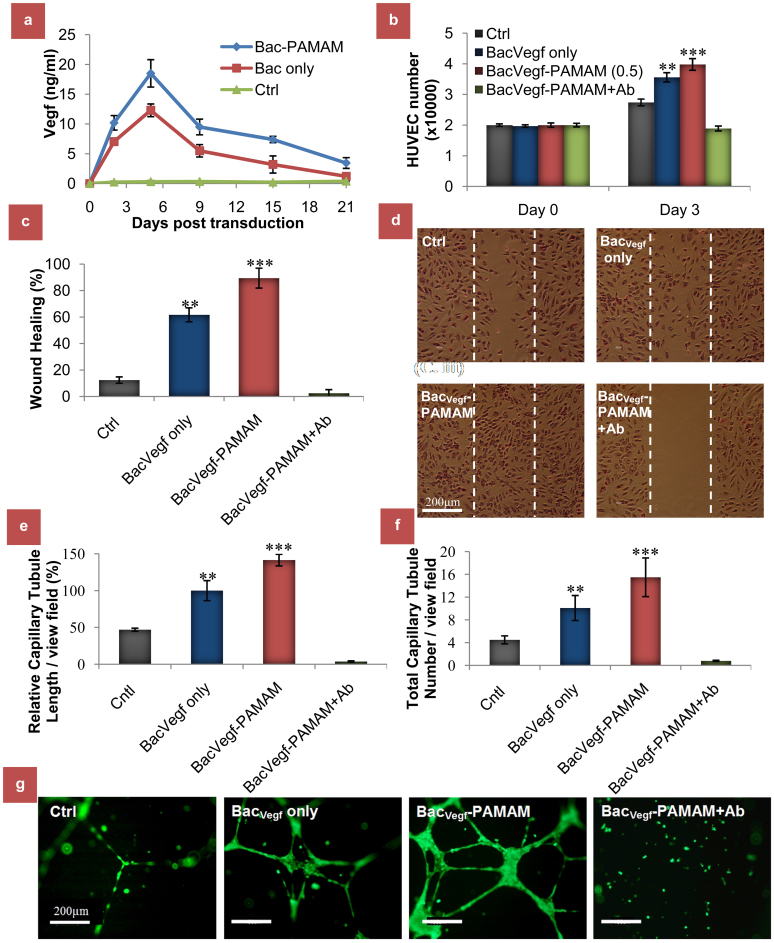
*In vitro* functional effects of bioactive stent on vascular endothelial cells. (a) Quantification of released Vegf from bioactive stent transduced HASMCs over time of three weeks. (b) Proliferation of HUVECs grown in the presence of CM from Bac_Vegf_-PAMAM (with or without Ab) and Bac_Vegf _transduced HASMCs. As control group, CM from non-transduced cells (Ctrl) was taken. Initial seeding density was 2 × 10^4^ cells per well in 96 well plate and cell proliferation was detected by a colorimetric assay on day3. (c) Induction of HUVEC migration in a wound healing assay. HUVEC cell monolayer was wounded with cell scraper and treated with CM from above mentioned groups. HUVEC were photographed (200×) after 24 h treatment (d) and percentage of scratched area (marked by the white dotted line) covered by the migrated cells were analyzed using Image J software. (e and f) Effect of CM from above mentioned groups on tubular formation *in vitro*. After 18 h co-cultivation with HUVECs, tubular formation was evaluated. The graphs represent the relative tubule length in μm taking Bac_Vegf _as 100% (e) and counting of capillary tubule number (f). The data represent the mean ± SD of three independent experiments. (g) Properly formed tubular structure was observed in Bac_Vegf_-PAMAM and Bac_Vegf_ groups, compared to the unstimulated control and Ab treated group as examined using a fluorescent microscope under 100× magnification. ANOVA: Significantly higher values in groups compared to control are denoted by *** = P < 0.001 and ** = P < 0.01.

To study the functionality of the Vegf proteins, expressed by the treated HASMC, different functionality assays were performed using in the conditioned media (CM) on human umbilical vein endothelial cells (HUVEC) which have high mitotic and chemotactic response to Vegf. Thus, CM were collected from Bac_Vegf_-PAMAM (0.5) and Bac_Vegf _transduced HASMCs at regular intervals, and used to quantify the Vegf release profile using Vegf ELISA analysis. CM from non-transduced cells was taken as the negative control. The data, as shown in Figure 2a, demonstrates rapid expressions of hVegf in Bac_Vegf_-PAMAM and Bac_Vegf _groups in the first 4 days which gradually decreased over the week. Although Vegf expression decreased considerably in Bac_Vegf _group after three weeks, Bac_Vegf_-PAMAM group maintained significantly higher Vegf expression. The bioactivities of the released Vegf in media from the Bac_Vegf_-PAMAM (0.5) transduced HASMC were evaluated *in vitro* by observing the proliferative capacity of the HUVEC. Cell Proliferation assay was used to assess the proliferation capabilities of the HUVEC treated with CM (containing secreted Vegf) from experimental samples, collected on day 4 post transduction. As shown in Figure 2b, groups Bac_Vegf_ and Bac_Vegf_-PAMAM showed significantly high HUVEC proliferation on day 3 compared to unstimulated control, with Bac_Vegf_-PAMAM exhibiting the highest proliferation (3.98 ± 0.19 × 10^4^ cells). As expected, group treated with antibodies against Vegf showed no proliferative effects proving that it was because of the bioactivity of released Vegf from genetically modified SMCs that contributed to the drastic HUVEC proliferations. This proliferation rate was directly dependant on the amount of the Vegf released which explains why Bac_Vegf_-PAMAM demonstrated better results than Bac_Vegf_ and unstimulated control groups.

Similar results were noticed in the wound healing assay where the abilities of CM from different experimental groups, collected on day 4 post transduction, to promote HUVEC migrations were measured. As depicted in Figure 2c–d, stimulation of wounded HUVEC monolayer with CM from Bac_Vegf_-PAMAM exhibited significant healing of wounded area (89.4 ± 7.5%) compared to CM from unstimulated control (12.4 ± 2.4%) and Bac_Vegf_ (61.7 ± 5.5%). Pre-incubation of CM with the neutralizing anti-Vegf antibodies completely hindered Bac_Vegf_-PAMAM CM induced wound healing, clearly suggesting that chemotactic signals from Vegf are required for proper wound healing effect.

In addition, the biologic activities of the CM were also evaluated by HUVEC tube formation assay. As illustrated in Figure 2e–g, cells treated with CM from Bac_Vegf_-PAMAM induced significantly enhanced effect on HUVEC capillary network formation as compared to the cells treated with CM from Bac_Vegf_ and unstimulated groups. The relative tubule length was significantly enhanced in Bac_Vegf_-PAMAM and Bac_Vegf_ group compared to control (141.5 ± 7.9 and 100 ± 13.6 vs 47 ± 2.1). Also, to assess the extent of impact of Vegf present in the CM on HUVEC tube formation, anti-Vegf antibody was added to the supernatant with released CM. A significantly reduced tube formation was observed which provides evidence of the strong pro-angiogenic nature of Vegf present in the CM. Similar results were also noticed when total capillary tubule numbers were quantified in Bac_Vegf_-PAMAM and Bac_Vegf_ group and compared to unstimulated control (15.5 ± 3.4 and 10.1 ± 2.2 vs 4.5 ± 0.7).

### *In vivo* efficacy of the bioactive stent in balloon denuded canine femoral artery

**Figure 3:**
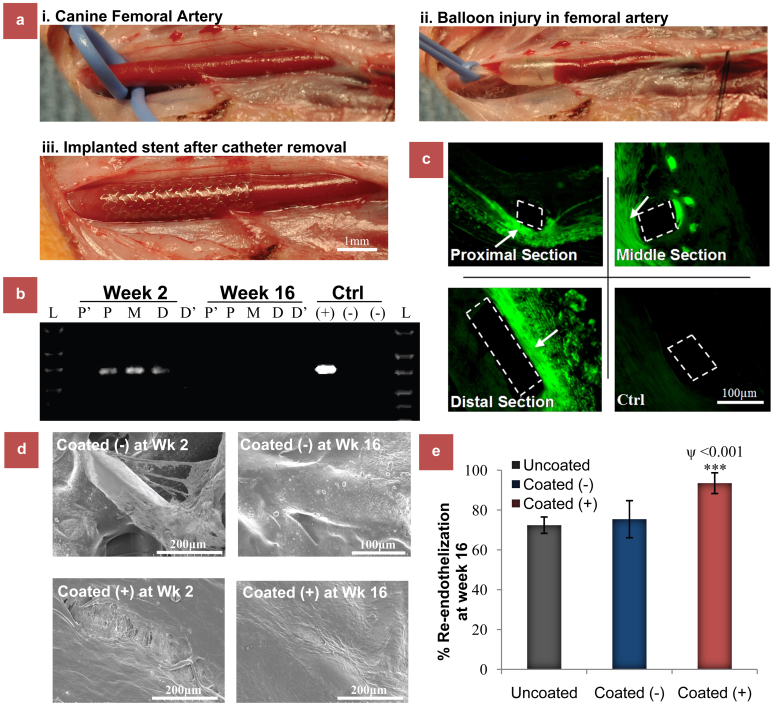
Arterial gene delivery and endothelial recovery of bioactive stents implanted in balloon injured dog femoral arteries. (a) Experimental procedure for the in vivo experiment in canine model. The femoral artery was first isolated from the normal blood flow using clips (a. i). The selected portion was then injured by balloon catheter (a. ii). This was followed by the implntation of the stent in the damaged artery (a. iii). (b) Vegf transgene delivery delivery and expression in the artery. RT-PCR of tissue retrieved from stented vessel segments was performed to identify the hVegf gene. 2 weeks post stent implantation in Coated (+) group, the Vegf transcript was still present in the proximal (P), middle (M) and distal (D) portions of the stented artery. But no gene delivery occured in the artery sections 1 cm proximal (P′) and distal (D′) to the stented portion. Importantly, the transcript also disappeared in Coated (+) group by week16 confirming the transient nature of expression. (c) Immunohistochemical localization of Vegf within stented femoral artery in Coated (+) group at week 2 post stent deployment. Coated (−) was taken as control. The Vegf expression occurred mainly at the strut area (white dotted) where the stent surface touched the inner lining of the artery, with no significant expression in the neointimal area indicating that the gene transfer from stent surface occurred only at the very early stage of deployment. (d) Re-endothelialization of vessels following stent implantation. SEM pictures of the stents on week 16. Vessels from Coated (−) group lacked endothelial cell morphology between struts, while endothelial cell monolayer completely covered the stent surface with typical fusiform morphology and intact borders in Coated (+) group. (e) Re-endothelialization of arterial tissue sections in different stents at week 16 post stent deployment by histological assessment of. The data represent the mean ± SD (n = 8). ANOVA: *** = P < 0.001 and * = P < 0.05, while P value on comparing Coated (+) and Coated (−) in (D) is denoted by ψ.

The *in vivo* stent implantation procedure has been demonstrated in Figure 3a, where the dogs underwent bilateral femoral artery denudation by balloon angioplasty, followed by stent deployment at the injured site^29^,^38^. Stented femoral arteries transduced with Coated (+) stent, carrying Bac_vegf_-PAMAM, Coated (−) stent carrying Bac_Null_-PAMAM, and uncoated bare metal stents (negative control) were harvested 14 days after stent placement. Following RT-PCR analysis, Vegf signal expression could be observed in all the proximal, middle and distal sections of the Bac_Vegf_-PAMAM [Coated(+)] transduced arteries examined (*n* = 3 stents), while nothing was detected in Bac_Null_-PAMAM [Coated (−)] transduced arteries (Figure 3b). Although, the RT-PCR product from 2 weeks Bac_Vegf_-transduced tissue samples demonstrated the presence of the appropriate-sized band for Vegf in the stented artery sections, no bands were detected from 16 weeks samples proving that the transgene expression is transient and disappears over time. Moreover, artery sections from 1 cm proximal and distal to the stented artery did not show any transgene expression, thus indicating that the gene delivery was localized and restricted to the stented region where the virus containing polymer coated strut touches the inner wall of the artery. The result was reconfirmed by immunostaining for expressed Vegf in the stented artery. This baculovirus nanohybrid mediated Vegf transgene expression was localized to the areas around the stent struts in the intimal and medial layers (Figure 3c), where the polymer of the stent touches the arterial surface; in contrast, there was no Vegf staining observed in the stented arteries treated with Coated (−) stents.

Endothelial regeneration was determined using three independent methodologies: Evan's blue staining, scanning electron microscopy and histology. Two weeks after balloon injury and stent placement, endothelial regeneration was assessed in the animals treated with Coated (+) and Coated (−) stents using Evans blue staining (*n* = 3 stent). Luminal staining for Evans blue demonstrated that the balloon angioplasty and stenting procedure completely denuded the femoral artery in Control (−) stent while Coated (+) showed marked recovery at week 2 (Figure S7a). As evident from Figure S7b, re-endothelialization was significantly greater in the Coated (+) vessels (55.36 ± 4.64%) in comparison with Coated (−) control vessels (37.5 ± 6.51%, *P* < 0.05).

Furthermore, at 2 weeks and 16 weeks after stent deployment, SEM pictures of the inner surface of the stented arteries were taken. Cells consistent with endothelial morphology were noted on the surface of Bac_Vegf_ treated stent struts on week 2, which completely covered the stent surface in a uniform way after 16 weeks (Figure 3d). In contrast, an irregular rough surface, mainly comprising of the exposed stent strut and partially covered neointima tissues, was noticed in the arteries with Bac_Vegf_ treated stent on both week 2 and week 16. Histological assessment of endothelial regeneration, sixteen weeks post stent placement, demonstrated a significant difference in endothelial regeneration between the three groups, Coated (+), Coated (−) and Uncoated bare metal negative control stents. The percentage of endothelial cells observed in the Coated (+) vessels was significantly higher than in the control vessels (93.5 ± 5.2% vs 75.4 ± 9.3% and 72.4 ± 4.1; *P* < 0.05) as shown in Figure 3e.

**Figure 4:**
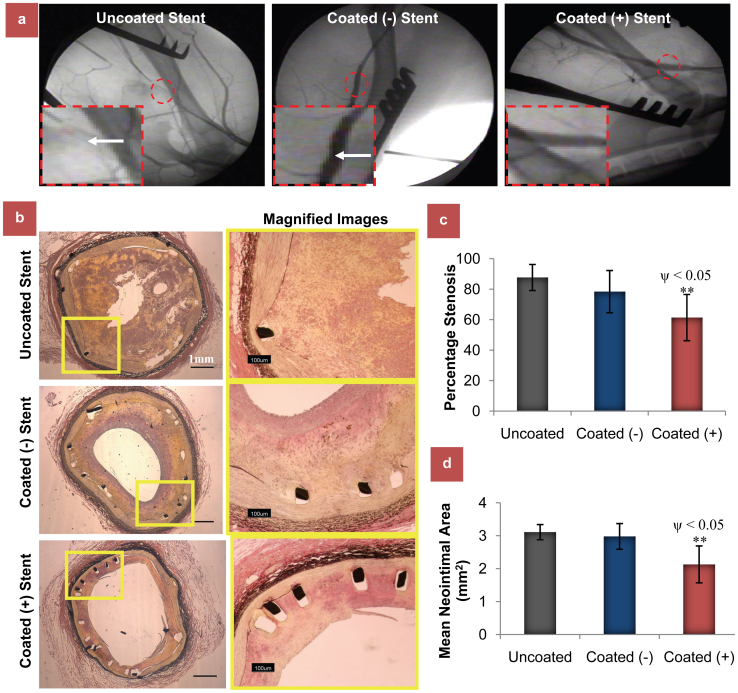
Assesment of *in vivo* biofunctionalility of the developed therapeutic bioactive stents. (a) The effect of Bac_Vegf_-PAMAM delivery on ISR assessed by angiography analysis. Comparison of angiography studies at week 16 after stent deployment in femoral arteries of dogs. Representative angiographic images of femoral arteries with uncoated bare metal stent and stents coated carrying Bac_Null_-PAMAM and Bac_Vegf_-PAMAM at week 16 after stent deployment, with white arrows showing the blockages (Supplementary Movies 1 and 2). (b) The effect of Bac_Vegf_-PAMAM delivery on ISR assessed by histomorphometric analysis. Comparison of histomorphometric studies at Week 16 after stent deployment in dog femoral arteries. Representative cross-sectional images of elastic Van Gieson stained femoral arteries with uncoated bare metal stent and stents coated carrying Bac_Null_-PAMAM and Bac_Vegf_-PAMAM at week 16 after stent deployment. Percentage stenosis (c) and mean neointimal area (d) analysis at the stented regions demonstrated significantly reduced ISR in Coated (+) group compared to Uncoated and Coated (−) groups. The data represent the mean ± SD (n = 8). ANOVA: ** = P < 0.01; P value on comparing Coated (+) and Coated (−) is denoted by ψ.

All vessels were angiographically and histologically patent throughout the period of study. Stent malapposition was also not detected in any animal. 16 weeks after site-specific Vegf gene transfer, the stenotic area was significantly reduced in Coated (+) compared to control uncoated group stents (54.58 ± 14.1% vs 85.4 ± 10.14%; *P* < 0.05) as analyzed by angiography (Figure 4a** and S7d**) using standard methods^39^. The extent of vessel injury at the stent site, as analysed by histological examinations^20^, was similar in all the groups as determined by injury score (Coated+ 1.13 ± 0.14 vs Coated- 1.15 ± 0.27 vs Uncoated 1.21 ± 0.35). Similar results were also obtained with inflammation score (Coated+ 0.66 ± 0.34 vs Coated- 0.7 ± 0.31 and Uncoated 0.81 ± 0.45; p > 0.05). Importantly, histomorphometric analysis (Figure 4b) also demonstrated a significantly reduced intimal hyperplasia or percentage stenosis in Coated (+) group compared to other two groups (Figure 4c, 61.36 ± 15.15% vs 78.41 ± 13.84 and 87.66 ± 8.54%; n = 8, *P* < 0.05). Similar results were obtained when analyzed in terms of cross-sectional mean neointimal area (Figure 4d, 2.23 ± 0.56 mm^2^ vs 2.78 ± 0.49 mm^2^ and 3.11 ± 0.23 mm^2^; p < 0.05). 1 cm proximal and distal to the stented area showed no signs of intima formation.

## Discussion

To the best of our knowledge, this study for the first time demonstrates that insect cell generated recombinant baculoviruses can be efficiently used for stent based gene therapy applications, such as inhibition of post angioplasty ISR. This work has focused on designing a new anti-restenosis strategy using a baculovirus based gene-eluting stent which ameliorates the biology of the vessel wall, in contrary to drug-based vascular disruption, by accelerating endothelial recovery after stent deployment. The data substantiate our hypothesis by demonstrating significantly improved endothelial recovery assessed by Evan's blue staining and scanning electron microscopy, and restricted ISR illustrated by histomorphometric and angiographic analysis over a study period of 4 months post implantation.

So far, baculoviruses have long been considered as a biologically safe gene delivery vector for mammalian cells^27^. Although it has been shown to effectively transduce mammalian cells *in vitro*, its full potential for *in vivo* gene therapy is not yet proven. Through this work, we have developed methods to protect it from host immune system as well as enhance its transduction ability which are its main limitations for *in vivo* applications. Moreover, its transient nature of recombinant gene expression is ideal in such a case where the expression ceases once the job is done. This also confirms the safety of the system to avoid unnecessary side effects in the post healing period. The availability of easily grown, suspension-ready host insect cells also facilitates the scale up process. As such, it is easier to obtain high titre of recombinant baculoviruses than other mammalian viruses. This makes the baculovirus expression system logistically beneficial and economically viable for process development and large production capacity.

This technology can be a potential alternative to the currently available DESs, which mainly use anti-proliferative drugs. These drugs, while preventing smooth muscle cell overgrowth, also lead to incomplete regeneration of the damaged endothelial layer. The implication of the newly formulated bioactive stent will enhance the Vegf based rapid re-endothelialization mechanism^40^ leading to better recovery process and reduced chance of restenosis. Moreover, the bioactivity of stent can be retained for at least 3 months once stored in the freezer, which further extends its commercial viability. In addition, the invention of the method documented here to effectively encapsulate functionally active baculoviruses also opens up the potential applications of recombinant baculoviruses for wider gene therapy applications. However, the present work does not demonstrate whether this system can inhibit long term in-stent thrombosis. An important area which needs further research is to characterize the *in vivo* dose dependant safety and efficacy analysis of the stent. In the present study we used a comparatively high dose of baculovirus nanohybrids. This is because, as a first study of its kind with no prior literature data, we aimed to achieve maximum transgene delivery at the stented site to achieve high growth factor expression, which can be a key determinant for the degree of vascular healing. Baculoviruses, with surface functionalized targeting ligands, can be the next step for this technology where the released viruses can site-specifically repair the denuded regions with minimal dosage. Furthermore, this advanced baculovirus based gene delivery system can be integrated with existing DES technology for combined stent based therapy of restenosis and thrombosis.

Taken together, the major findings of the current work capitalize on the development of this novel approach where local delivery of Bac_Vegf_-PAMAM nanobiohybrid vector, entrapped within PLGA MSs embedded layer-by-layer on fibrin-coated stent, can significantly enhance endothelial recovery and thereby block subsequent neointimal proliferation and ISR. Importantly, this transient nature of insect cell-originated baculovirus nanohybrid gene delivery system makes it a prospective biologically safe material for advanced translational research.

## Methods

### Generation of recombinant baculoviruses

The recombinant Vegf baculoviruses (Bac_Vegf_) were generated by cotransfection of Sf9 insect cells with linearized baculovirus DNA (BD Baculogold) along with purified recombinant transfer vectors using cellfectin (Invitrogen Life Technologies, Carlsbad, CA) transfection reagent as mentioned in earlier study^24^. The viral titre [plaque forming unit (pfu)/mL] of the amplified viral stock was then determined using the Baculovirus Fast Plax Titer Kit (Novagen, Madison, WI) according to the manufacturer's protocol. Similarly, baculoviruses carrying MGFP gene (Bac_MGFP_) and no transgene (Bac_Null_) were also generated.

### Formulation of bac-PAMAM nanohybrids

PAMAM dendrimer (generation 0) with ethylenediamine core (MW: 516.68) containing 4 surface primary amino groups was procured from Sigma Chemicals and resuspended in phosphate buffered saline (PBS). The concentration of PAMAM peptide stock solution was initially adjusted to 10 μmol in PBS solution. In order to form the PAMAM-baculovirus nanocomplex, the solutions of the PAMAM and baculoviruses were first brought to room temperature and adjusted to desired concentrations. Then the PAMAM solution and baculovirus solution were mixed according to a desired PAMAM/virus ratio (0, 0.01 μmol, 0.1 μmol, 0.5 μmol and 1.0 μmol PAMAM molecules per 10^8^ baculovirus). The mixture was incubated at room temperature for 30 min to form complexes, with gentle vortexing from time to time. The mixture was further centrifuged at 24,000 rpm for 45 min, and the pellet containing the heavier Bac-PAMAM nanocomplex was collected, leaving the unreacted excess dendrimer in the supernatant. The collected Bac-PAMAM was washed twice using PBS using the same centrifugation process. For every experiment, the preparation was freshly made.

### Preparation of bioactive microparticles containing baculovirus nanohybrids

In order to encapsulate the the viral particles by water-oil-oil (w/o/o) double emulsion and solvent evaporation method^34^, 5 × 10^13^ pfu was resuspended in 100 μl of PBS containing 10% glycerol and 50 mg/ml BSA. The primary waterr in oil emulsion solution was prepared by homogenizing it in 1 ml of DCM containing 50 or 100 mg of PLGA [poly(D,L-lactic-co-glycolic acid)] and 1 ml acetonitrile for 1 min at 10,000 rpm. The resulting primary emulsion was added to 5 ml of corn oil containing 2% span80 and homogenised for 3 min at 14000 rpm to form the secondary w/o/o emulsion. This solution was further agitated with a magnetic stirring bar in 10 ml of of corn oil containing 2% span80 for 4 h to evaporate the the DCM. The hardened PLGA microspheres were centirfuged at 9000 g for 10 min, washed thrice with PBS and stored temporarily at 4°C. In a similar way, PLGA microparticles were prepared by water-oil-water (w/o/w) method^34^. The method of PLGA microencapsulation of virus particles are demonstrated schematically in Figure S2.

### Preparation of bioactive stent using layer-by-layer coating

Firstly, the baculovirus was coated with PAMAM (0.5 μmol) and formulated in PLGA microspheres by w/o/o method. The prepared PLGA microspheres were resuspended in 5 mg/ml of bovine plasma derived fibrinogen, supplemented with aprotinin (20 μg/ml) to reduce fibrin degradation, and loaded in a 3 ml syringe with a 0.2-mm nozzle. The balloon expandable bare metal stainless steel stents with basic dimensions of 16 mm × 3.5 mm (Liberte Monorail stent, Boston Scientific, Mississauga, Ontario) was first mounted on a PTFE (polytetrafluoroethylene) mandrel that was driven by a rotator. The loaded aqueous fibrinogen mixture in the syringe was then gradually drip-coated on the surface of the mounted stent layer by layer (0.2 ml of Bac loaded aqueous fibrinogen per layer). In between every layer, 0.05 ml of thrombin solution (20 U/ml) was added all over the stent surface using a micropipette with 200 μl micropipette tip and waited for 15 min to form thin fibrin gel layer. Polymerization of the fibrin occurred around the stent which completely encased the stent (Figure S1). The stent was subsequently coated with a top layer of fibrinogen (2.5 mg/ml) cross-linked with genipin (0.045 mg/mL final) followed by polymerization with thrombin. The process resulted in a microsphere impregnated fibrin coated stent loaded with 5 × 10^12^ pfu Bac. The device was then crimped onto standard collapsed angioplasty balloon and delivered to the femoral artery. [The detailed methods for *in vitro* transduction and biofunctional studies are present in the Supplementary information.]

### *In vivo* surgical procedures for arterial injury and stent implantation

All procedures were in compliance with the Canadian Council on Animal Care and McGill University animal use protocol, following all the ethical guidelines for experimental animals. Adult beagle dogs (Marshall Farms, North Rose, NY) weighing 9.5 to 11 kg were used in this study. A total of 30 stents were implanted bilaterally in deep femoral arteries of 15 dogs in a randomized, blinded fashion following denudation of the arterial endothelial wall, with contralateral arteries receiving stents from different groups. The animals were divided into three groups: fibrin coated stent loaded with microencapsulated PAMAM-Bac_Vegf _virus [Coated (+); n = 11 stents per group)], fibrin coated stent loaded with microencapsulated PAMAM-Bac_Null_ virus [Coated (−); n = 11 stents per group] and bare metal stent (Uncoated; n = 8 stents per group). To confirm the transgene delivery and re-endothelization, animals from first two groups were sacrificed (n = 3) after 2 weeks and arteries were harvested. The remaining animals were sacrificed after 16 weeks for further analysis.

Denudation of the femoral arterial endothelial layer and stent implantation was performed according to the procedure performed in earlier studies^38^. The selected portion of artery lumens were flushed with PBS to avoid mixture with arterial blood. On the basis of angiograms, femoral segments with comparable diameters were selected on both legs so that the stent-to-artery diameter ratio remains approximately 1.3. After an arteriotomy, a fogarty arterial embolectomy balloon catheter (Edwards Lifesciences Canada Inc, Ontario) was infused through the saphenous arteries and advanced to the two preselected femoral segments and secured with a tie as mentioned elsewhere^29^. This was followed by severe balloon injury of the inner lumen of the artery with inflated balloon (balloon/artery ratio 1.2:1) to induce endothelial abrasion. Eventually the balloon catheter mounted stents were deployed at the site. 3 animals were euthanized at week 2, while the remaining at week 16.

### Analysis of transgene expression

#### RT-PCR analysis

At week 2 post stent placement, the stented femoral arteries (n = 3 stents/group) were harvested from Coated (+) and Coated (−) groups and divided laterally into three sections (proximal, mid and distal) after removing the stents. A part of the sections were used to detect hVegf transcript by RT-PCR, while the remaining parts were used for to detect the hVegf protein by immunostaining and reendothelization by staining. Similarly, sections were also collected at week 16. PCR amplification was performed on the reverse transcribed product using Taq DNA Polymerase (Invitrogen) and forward primer (5′CTTGCCTTGCTGCTCTACCTCC3′) and reverse primer (5′GCTGCGCTGATAGACATCCATG3′) for hVegf gene (112-bp product). Amplifications were carried out for 25 cycles at 94°C for 35 s (denaturation), 57°C for 35 s (annealing), and 72°C for 25 s (extension).

#### Immunohistochemical studies

For immunostaining rabbit anti-hVegf (Santa Cruz Biotechnology Inc., Santa Cruz, California) primary antibodies were used. Donkey anti-rabbit IgG-FITC was used as secondary antibody. The proportions and intensities of FITC-positive regions in the tissue sections, as seen under fluorescence microscope, gave a qualitative idea of the relative amount of hVegf expressed in the stented vascular tissue regions due to the transgene delivery from stent platform.

### Assessment of stent re-endothelialization

#### Evans blue staining

Two weeks after stent deployment animals were anesthetized [*n* = 3, Coated (+) and (−)] and a portion of the harvested artery was incubated in 1% Evans blue (Sigma-Aldrich) for 15 min, followed by washing ,fixing and examining longitudinally using image software as mentioned elsewhere^20^.

#### Scanning electron microscope

Re-endothelialization was also assessed at 2 week and 16 week post implantation (*n* = 3 each). Retrieved stents were washed with saline, fixed in 4% paraformaldehyde and longitudinally incised as above and examined using scanning electron microscopy (SEM).

#### Histological assessment

Stents were retrieved after twelve weeks and harvested vessels were embedded in methylmethacrylate plastic (Accel Lab Inc, Quebec, Canada and McGill SAIL Lab). After polymerization, the proximal, mid and distal sections of the stents were cut into 5 μm sections and stained with hematoxylin and eosin. Re-endothelialization was assessed directly under the microscope by a histologist blinded to treatment. Endothelial coverage was expressed as the percentage of the average lumen circumference covered by the endothelial cells^20^.

### Assessment of ISR

#### Angiogram analysis

Initial and follow-up angiograms were performed with fluoroscopic angiography (GE Stenoscop) using Omnipaque Iohexol contrast dye in anterior oblique projection and percent diameter stenosis at follow-up was calculated by (minimal stent diameter at follow-up/the mean diameter of the stent at full expansion) × 100, using standard procedure as described elsewhere^39^. Sections were also used to evaluate the presence of inflammation and vessel wall injury at the stented sites of all the groups by injury and inflammation score method^20^,^39^.

#### Morphometric analysis

Retrieved vessels were embedded in methylmethacrylate plastic, cut into thin sections as mentioned earlier, and stained with elastic Van Gieson stain. All sections were examined by light microscopy and photographed to quantify the neointima formation and stenotic area using methods mentioned elsewhere^20^.

### Statistical analysis

Quantitative variables are presented as mean ± Standard Deviation (SD) from independent experiments as described in the figure legends. Statistics were performed using student's t-test or one-way ANOVA by Bonferroni's multiple comparison post-hoc test. All statistical analyses were performed with Prism 5 (GraphPad Software). P value <0.05 was considered significant.

## Author Contributions

A.P., C.B.E. and S.P. designed the study. A.P. and D.S.T. (cardiac surgeon) performed the animal experiments. A.P., D.S.T. and S.P. analyzed the results. C.B.E. provided technical insights, assisted in virus production and investigated the cell-nanohybrid virus interactions. A.P. and S.P. wrote the paper. The manuscript was revised by all the authors and has approved to the final version of the manuscript.

## Supplementary Material

Supplementary Information Supplementary Information

## References

[b1] Cully M. Treating restenosis after drug-eluting stent implantation. Nat Rev Cardiol 10, 62 (2013). 23247311 10.1038/nrcardio.2012.189

[b2] van der Heiden K. *et al.* The effects of stenting on shear stress: relevance to endothelial injury and repair. Cardiovasc Res (2013). 10.1093/cvr/cvt090 23592806

[b3] Dangas G. D. *et al.* In-stent restenosis in the drug-eluting stent era. J Am Coll Cardiol 56, 1897–1907 (2010). 21109112 10.1016/j.jacc.2010.07.028

[b4] Paul A., Shao W., Shum-Tim D. & Prakash S. The attenuation of restenosis following arterial gene transfer using carbon nanotube coated stent incorporating TAT/DNA(Ang1 + Vegf) nanoparticles. Biomaterials 33, 7655–7664 (2012). 22818986 10.1016/j.biomaterials.2012.06.096

[b5] Chan J. M. *et al.* In vivo prevention of arterial restenosis with paclitaxel-encapsulated targeted lipid-polymeric nanoparticles. Proc Natl Acad Sci U S A 108, 19347–19352 (2011). 22087004 10.1073/pnas.1115945108 PMC3228458

[b6] Langer R. & Tirrell D. A. Designing materials for biology and medicine. Nature 428, 487–492 (2004). 15057821 10.1038/nature02388

[b7] Prakash S., Malhotra M., Shao W., Tomaro-Duchesneau C. & Abbasi S. Polymeric nanohybrids and functionalized carbon nanotubes as drug delivery carriers for cancer therapy. Adv Drug Deliv Rev 63, 1340–1351 (2011). 21756952 10.1016/j.addr.2011.06.013

[b8] Binsalamah Z. M., Paul A., Prakash S. & Shum-Tim D. Nanomedicine in cardiovascular therapy: recent advancements. Expert Rev Cardiovasc Ther 10, 805–815 (2012). 22894635 10.1586/erc.12.41

[b9] Shuchman M. Debating the risks of drug-eluting stents. N Engl J Med 356, 325–328 (2007). 17251527 10.1056/NEJMp068300

[b10] Iakovou I. *et al.* Incidence, predictors, and outcome of thrombosis after successful implantation of drug-eluting stents. JAMA 293, 2126–2130 (2005). 15870416 10.1001/jama.293.17.2126

[b11] Mitragotri S. & Lahann J. Materials for drug delivery: innovative solutions to address complex biological hurdles. Adv Mater 24, 3717–3723 (2012). 22807037 10.1002/adma.201202080

[b12] Holmes D. R. Jr Incidence of late stent thrombosis with bare-metal, sirolimus, and paclitaxel stents. Rev Cardiovasc Med 8 Suppl 1, S11–18 (2007). 17401306

[b13] Goh D. *et al.* Nanotechnology-based gene-eluting stents. Mol Pharm 10, 1279–1298 (2013). 23394068 10.1021/mp3006616

[b14] Tan A. *et al.* Inception to actualization: next generation coronary stent coatings incorporating nanotechnology. J Biotechnol 164, 151–170 (2013). 23376617 10.1016/j.jbiotec.2013.01.020

[b15] Neumeister A. *et al.* Interface of nanoparticle-coated electropolished stents. Langmuir 28, 12060–12066 (2012). 22834824 10.1021/la300308w

[b16] Saurer E. M. *et al.* Polyelectrolyte Multilayers Promote Stent-Mediated Delivery of DNA to Vascular Tissue. Biomacromolecules (2013). 10.1021/bm4005222 PMC3683994 23597075

[b17] Nakano K. *et al.* Formulation of nanoparticle-eluting stents by a cationic electrodeposition coating technology: efficient nano-drug delivery via bioabsorbable polymeric nanoparticle-eluting stents in porcine coronary arteries. JACC Cardiovasc Interv 2, 277–283 (2009). 19463437 10.1016/j.jcin.2008.08.023

[b18] Zhu D. *et al.* Local gene delivery via endovascular stents coated with dodecylated chitosan-plasmid DNA nanoparticles. Int J Nanomedicine 5, 1095–1102 (2010). 21270960 10.2147/IJN.S14358 PMC3023238

[b19] Gaharwar A. K. *et al.* Bioactive Silicate Nanoplatelets for Osteogenic Differentiation of Human Mesenchymal Stem Cells. Adv Mater (2013). 10.1002/adma.201300584 23670944

[b20] Sharif F. *et al.* Gene-eluting stents: adenovirus-mediated delivery of eNOS to the blood vessel wall accelerates re-endothelialization and inhibits restenosis. Mol Ther 16, 1674–1680 (2008). 18714308 10.1038/mt.2008.165

[b21] Yang J. *et al.* The prevention of restenosis in vivo with a VEGF gene and paclitaxel co-eluting stent. Biomaterials 34, 1635–1643 (2013). 23199742 10.1016/j.biomaterials.2012.11.006

[b22] Hiltunen M. O. *et al.* Intravascular adenovirus-mediated VEGF-C gene transfer reduces neointima formation in balloon-denuded rabbit aorta. Circulation 102, 2262–2268 (2000). 11056103 10.1161/01.cir.102.18.2262

[b23] Paul A., Shao W., Abbasi S., Shum-Tim D. & Prakash S. PAMAM dendrimer-baculovirus nanocomplex for microencapsulated adipose stem cell-gene therapy: in vitro and in vivo functional assessment. Mol Pharm 9, 2479–2488 (2012). 22817267 10.1021/mp3000502

[b24] Paul A. *et al.* A nanobiohybrid complex of recombinant baculovirus and Tat/DNA nanoparticles for delivery of Ang-1 transgene in myocardial infarction therapy. Biomaterials 32, 8304–8318 (2011). 21840594 10.1016/j.biomaterials.2011.07.042

[b25] Paul A., Nayan M., Khan A. A., Shum-Tim D. & Prakash S. Angiopoietin-1-expressing adipose stem cells genetically modified with baculovirus nanocomplex: investigation in rat heart with acute infarction. Int J Nanomedicine 7, 663–682 (2012). 22334788 10.2147/IJN.S26882 PMC3278230

[b26] Shiojima I. & Walsh K. The role of vascular endothelial growth factor in restenosis: the controversy continues. Circulation 110, 2283–2286 (2004). 15492329 10.1161/01.CIR.0000146723.23523.47

[b27] Lesch H. P. *et al.* Requirements for baculoviruses for clinical gene therapy applications. J Invertebr Pathol 107 Suppl, S106–112 (2011). 21784225 10.1016/j.jip.2011.05.010

[b28] Kaikkonen M. U., Maatta A. I., Yla-Herttuala S. & Airenne K. J. Screening of complement inhibitors: shielded baculoviruses increase the safety and efficacy of gene delivery. Mol Ther 18, 987–992 (2010). 20179675 10.1038/mt.2010.25 PMC2890102

[b29] Newman K. D. *et al.* Adenovirus-mediated gene transfer into normal rabbit arteries results in prolonged vascular cell activation, inflammation, and neointimal hyperplasia. J Clin Invest 96, 2955–2965 (1995). 8675667 10.1172/JCI118367 PMC186007

[b30] Kim Y.-K., Jiang H.-L., Je Y.-H., Cho M.-H. & Cho C.-S. Modification of baculovirus for gene therapy. Communicating Current Research and Educational Topics and Trends in Applied Microbiology *Vol. II*, 875–884 (2007).

[b31] van Oers M. M. Opportunities and challenges for the baculovirus expression system. J Invertebr Pathol 107 Suppl, S3–15 (2011). 21784228 10.1016/j.jip.2011.05.001

[b32] Balmert S. C. & Little S. R. Biomimetic delivery with micro- and nanoparticles. Adv Mater 24, 3757–3778 (2012). 22528985 10.1002/adma.201200224 PMC3627374

[b33] Soike T. *et al.* Engineering a material surface for drug delivery and imaging using layer-by-layer assembly of functionalized nanoparticles. Adv Mater 22, 1392–1397 (2010). 20437489 10.1002/adma.200903069

[b34] Matthews C., Jenkins G., Hilfinger J. & Davidson B. Poly-L-lysine improves gene transfer with adenovirus formulated in PLGA microspheres. Gene Ther 6, 1558–1564 (1999). 10490765 10.1038/sj.gt.3300978

[b35] McKenna C. J. *et al.* Fibrin-film stenting in a porcine coronary injury model: efficacy and safety compared with uncoated stents. J Am Coll Cardiol 31, 1434–1438 (1998). 9581746 10.1016/s0735-1097(98)00080-1

[b36] Slaughter B. V., Khurshid S. S., Fisher O. Z., Khademhosseini A. & Peppas N. A. Hydrogels in regenerative medicine. Adv Mater 21, 3307–3329 (2009). 20882499 10.1002/adma.200802106 PMC4494665

[b37] Macaya D., Ng K. K. & Spector M. Injectable Collagen–Genipin Gel for the Treatment of Spinal Cord Injury: In Vitro Studies. Advanced Functional Materials 21, 4788–4797 (2011).

[b38] Welt F. G., Edelman E. R., Simon D. I. & Rogers C. Neutrophil, not macrophage, infiltration precedes neointimal thickening in balloon-injured arteries. Arterioscler Thromb Vasc Biol 20, 2553–2558 (2000). 11116052 10.1161/01.atv.20.12.2553

[b39] Schwartz R. S. *et al.* Restenosis and the proportional neointimal response to coronary artery injury: results in a porcine model. J Am Coll Cardiol 19, 267–274 (1992). 1732351 10.1016/0735-1097(92)90476-4

[b40] Montanez R., Sanchez-Jimenez F., Quesada A. R. & Medina M. A. Exploring and challenging the network of angiogenesis. Sci Rep 1, 61 (2011). 22355580 10.1038/srep00061 PMC3216548

