# Supplementary material for: Bioactive baculovirus nanohybrids for stent based rapid vascular re-endothelialization
Source: Sci Rep. 2013 Aug 6;3:2366. doi: 10.1038/srep02366 (PMC3734445; doi:10.1038/srep02366)
Supplement: Supplementary Information [file srep02366-s1.pdf]

Supporting Information

Bioactive baculovirus nanohybrids for stent based rapid vascular re-endothelialization

Arghya Paul[1], Cynthia Elias[2], Dominique Shum-Tim[3], Satya Prakash[1]*

[1] Biomedical Technology and Cell Therapy Research Laboratory,
Department of Biomedical Engineering,
Faculty of Medicine, McGill University, 3775 University Street, Montreal,
Quebec H3A 2B4, Canada.

[2] Bulk Manufacturing, Sanofi Pasteur, Connaught Campus, 1755 Steeles Avenue West, Toronto, Ontario, M2R 3T4, Canada.

[3] Divisions of Cardiac Surgery and Surgical Research, The Montreal General Hospital,
1650 Cedar Ave, Suite C9-169, Montreal,
Quebec H3G 1A4, Canada.

*Correspondence author: Satya Prakash
Email: satya.prakash@mcgill.ca
Tel: +1-514-398-3676
Fax: +1-514-398-7461

Keyword: Nanomaterials, Hydrogel, Tissue engineering, Biotherapeutics, Stents, Endothelial cells.

Methods

In vitro transduction experiments using the developed bioactive stent

The stents with different formulations were crimped on the balloon catheter and inflated in scintillation vials containing 5ml of phosphate buffer saline (PBS) solution (pH 7.4) with 9 atm pressure. The expanded stent was incubated at 37°C for 24h, with constant agitation at 100 rpm. The PBS solutions containing the released viruses were collected at 12h, 18h and 24h post incubation.

The 12h incubation buffer was diluted and added at MOI of 250 to 1x10^{6} seeded human aortic artery smooth muscle cells (HASMCs; Sciencell, Carlsbad, California, USA) after removing the standard smooth muscle cell media (with no supplemented Vegf in it). The 18h and 24h incubation buffers were similarly diluted with same dilution factor and added to HASMCs. After incubating the cells with the stent incubation buffer for 8h, the buffers were aspirated and replenished with complete growth medium for 24h. After fixation, microphotographs were taken and the total cell numbers were determined per 200X magnification under bright field. GFP-expressing cells were also visually counted in the same fields, and results reported as the percentage of cells transduced (mean±SD) of at least five fields per culture in triplicate cultures.

In vitro transduction via Bac$_{Vegf}$-PAMAM loaded bioactive stent

Vegf release kinetics from transduced cells: In order to investigate the Vegf release kinetics from transduced HASMCs, three types of stents (Bac$_{Vegf}$-PAMAM, Bac$_{Vegf}$ only and ctrl stent with no virus) were expanded in PBS solution using balloon catheter and incubated for 24h as illustrated earlier. The PBS solution containing the released viruses were collected and added to 1x10^{6} HASMCs seeded per well in 6 well plate with an MOI 500. This was followed by 8h incubation at 25°C with subsequent replenishment of the incubated cells with fresh culture media. The conditioned media were collected on day 0, 2, 4, 9, 12 and 15 post-transduction and quantified for Vegf expression using hVegf ELISA kit (R&D Systems).

HUVEC proliferation assay: For the cell proliferation assay, 2x10^{4} Human Umbilical Endothelial Cells (HUVECs) /well were seeded in triplicate for each sample in 96-well plates. After culturing for 8h in standard endothelial cell media (ECM), the cells were washed twice with PBS. 0.1ml of CM from transduced HASMCs (Day 4 CM from Bac$_{Vegf}$-PAMAM, Bac$_{Vegf}$ only and ctrl stent with no virus groups) with and without hVegf

antibodies (Ab) along with 0.1 ml of fresh ECM without cell growth supplements were added to the corresponding set of wells. Similarly, CM from unstimulated control group mixed with fresh ECM was taken as the control group. After 3 days of culture, absorbance was measured at 490nm using Cell Titer 96 Aqueous Non-Radioactive Cell Proliferation Assay (Promega) in a plate reader as mentioned in previous study. This assay was also used to measure the cytototoxic effects of stent-released baculoviruses towards HASMCs.

Wound healing assay: HUVECs were seeded into six well plates and grown to confluency. After 24 hr of serum starvation, the monolayer was carefully mechanically wounded with a 200ul pipette tip. The wells were then washed twice with PBS to remove the cell debris and the seeded cells were replenished with 0.1 ml of the fresh ECM (without cell growth supplements) and 0.1ml of the CM from different groups (CM from day4 Bac_{Vegf}, Bac_{Vegf}-PAMAM and ctrl unstimulated group), in presence or absence of 1µg/mL Vegf antibody. Following HUVEC migration for 12h, the cells were fixed with 4% paraformaldehyde and stained with crystal violet. The wound healing was visualized under inverted bright field microscope and microphotographs with 100X magnification were taken. The number of cells which had moved across the starting line (mean ± SD; n=3) in each group was assessed and analyzed using Image J software to measure the wound healing as mentioned earlier.

HUVEC Tube formation Assay: In vitro angiogenesis assay was performed using Cell Biolabs Endothelial Tube Formation Assay as mentioned in our earlier studies. Briefly, 50µl of ECM gel prepared from Engelbreth–Holm–Swarm (EHS) tumor cells were added to the 96well plate and incubated for 1 h at 37°C to allow the gel to solidify. 2x10^{4} cells suspended in CM from different groups (Day 4 CM from Bac_{Vegf}-PAMAM, Bac_{Vegf} only and ctrl stent with no virus groups), in presence or absence of Vegf antibody were seeded per well. After 18h incubation period, media were removed and the cells were then incubated with 50µL of 1X Calcein AM for 30 mins at 37°C. The cells were washed twice with 1X PBS and the endothelial capillary-like tube formation in each well was examined using a fluorescent microscope under 100X magnification and the HUVEC-made capillary network was analyzed by Image J software. The results were quantified as the mean relative tubule length/view field ± SD, taking total tubule length/view field from control group as 100.

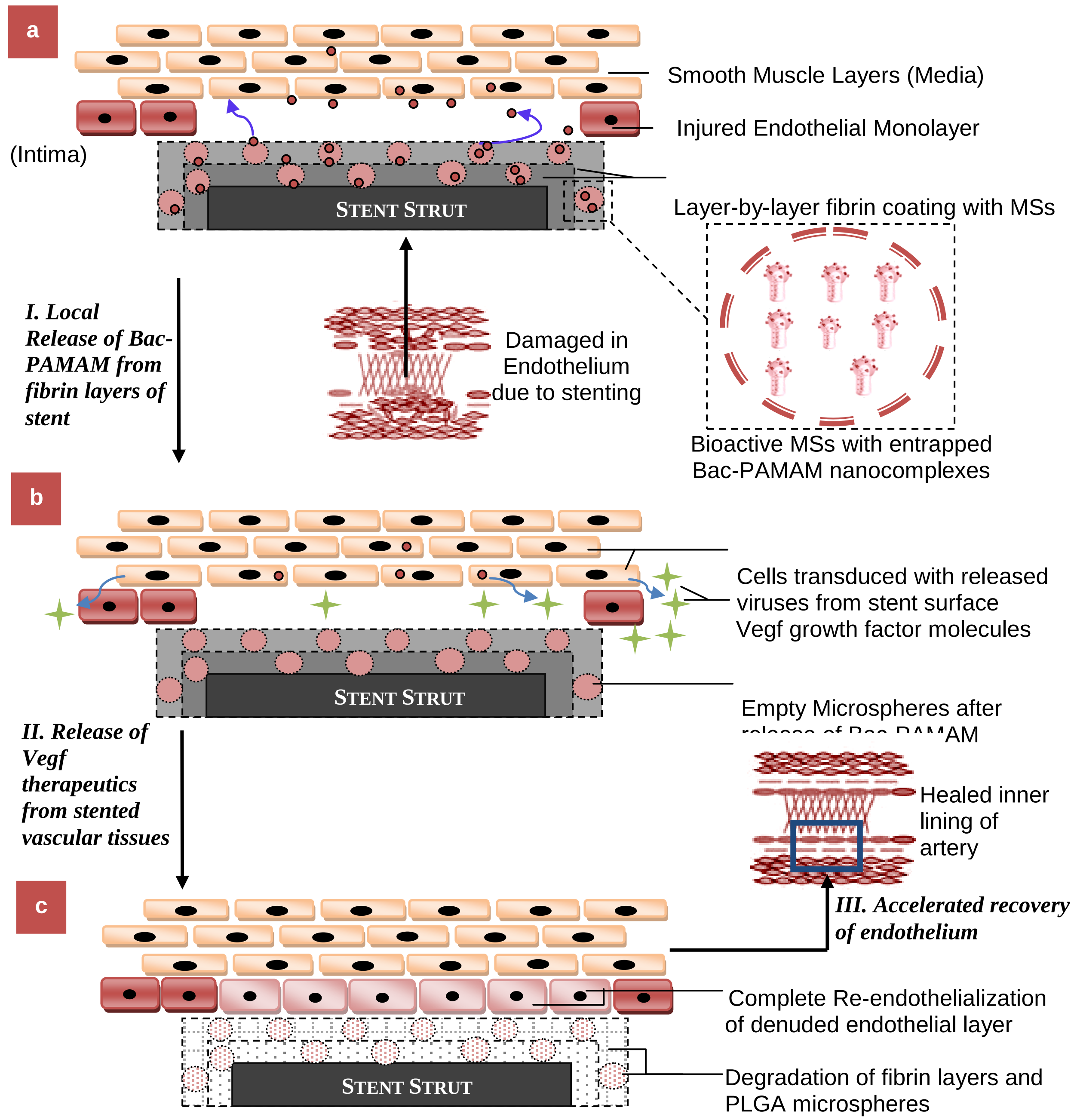

Figure S1: Schematic representation of design, formulation and mode of action of bioactive virus nanohybrid loaded stent for vascular endothelial injury therapy and attenuation of ISR. A. Release of entrapped viruses carrying angiogenic transgene (Vegf) to the adhering vascular wall. B. Local overexpression of transgene to enhance endothelial regeneration. C. Proper cushioning of intima layer with regenerated endothelial monolayer to reduce risk of restenosis by smooth muscle proliferation and thrombosis by avoiding further exposure to blood. With time, the stent loses its coatings due to the biodegradable nature of the polymers, leaving behind the bare stent struts in the already recovered vascular segment. On a separate note, re-endothelialization also occurs on the luminal surface of the stent which is not shown the figure.

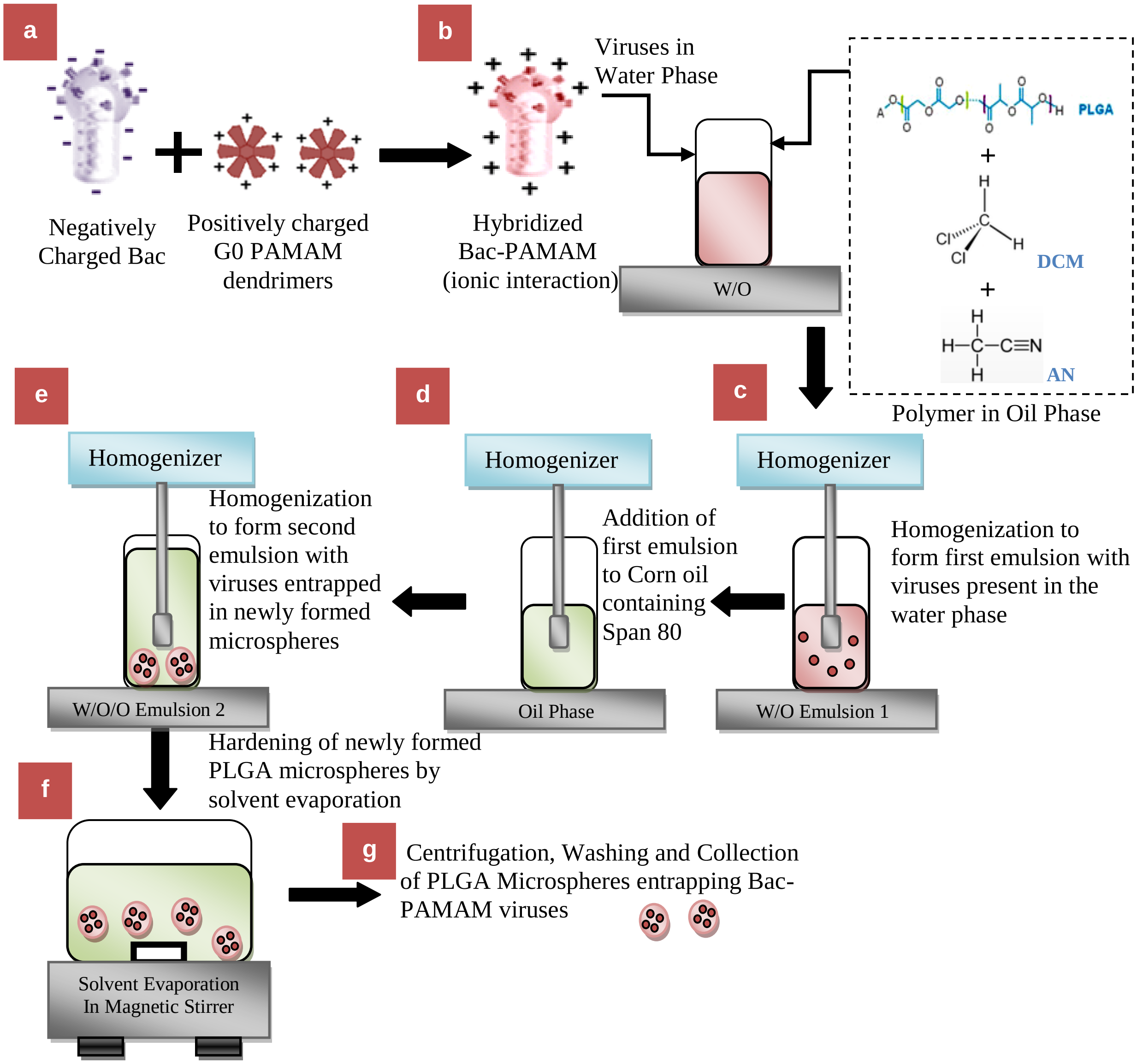

Figure S2: Schematic representation Bioactive Microparticle preparation. Generation of Bac-PAMAM complex and its subsequent microencapsulation in PLGA microspheres by water in oil in oil (w/o/o) double emulsion solvent evaporation method.

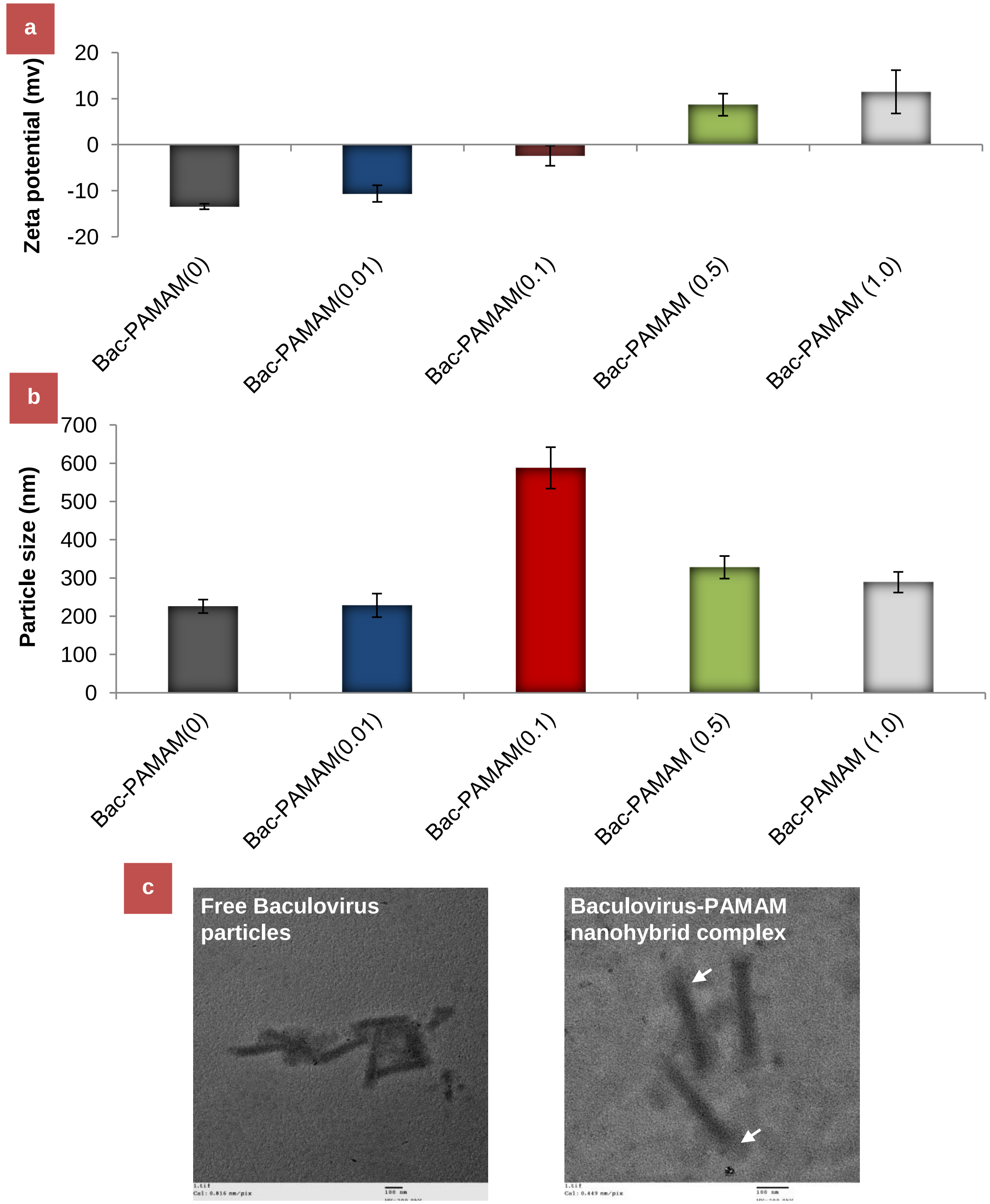

Figure S3: Surface modification of baculovirus with PAMAM dendrimer (G0). Zeta potential (a) and particle size (b) of Bac-PAMAM (0) (free Bac), Bac-PAMAM (0.01), Bac-PAMAM (0.1), Bac-PAMAM (0.5), and Bac-PAMAM (1.0), where the values within brackets indicate the ratio of PAMAM molecules in µmol per 10^8 viruses. The data is represented by mean ± standard deviation (SD). (c) TEM images of free Bac and Bac-PAMAM (0.5) suspended in PBS. Arrows indicate the positively charged PAMAM dendrimer coating on the negatively charged baculovirus surfaces to form the hybridized nanostructures. Scale indicates 100nm length.

Table S1: Effect of PLGA MS preparation procedure on active virus encapsulation efficiency (in terms of percentage of initial loaded viral titre).

Method	Homogenization		Sonication	
	Bac	***Bac+ BSA/glycerol***	***Bac***	***Bac+ BSA/glycerol***
**w/o/w (% pfu)**	15.5±0.7	35.6±3.1	10.6±1.3	26.4±4.1
**Diameter (μm)**	7.2±2.3	9.8±3.4	5.2±3.4	5.6±4.2
**w/o/o (% pfu)**	21.0±1.1	41.4±2.2	17.8±1.0	26.8±3.5
**Diameter(μm)**	9.2±2.6	8.3±3.2	5.6±1.1	4.9±2.2

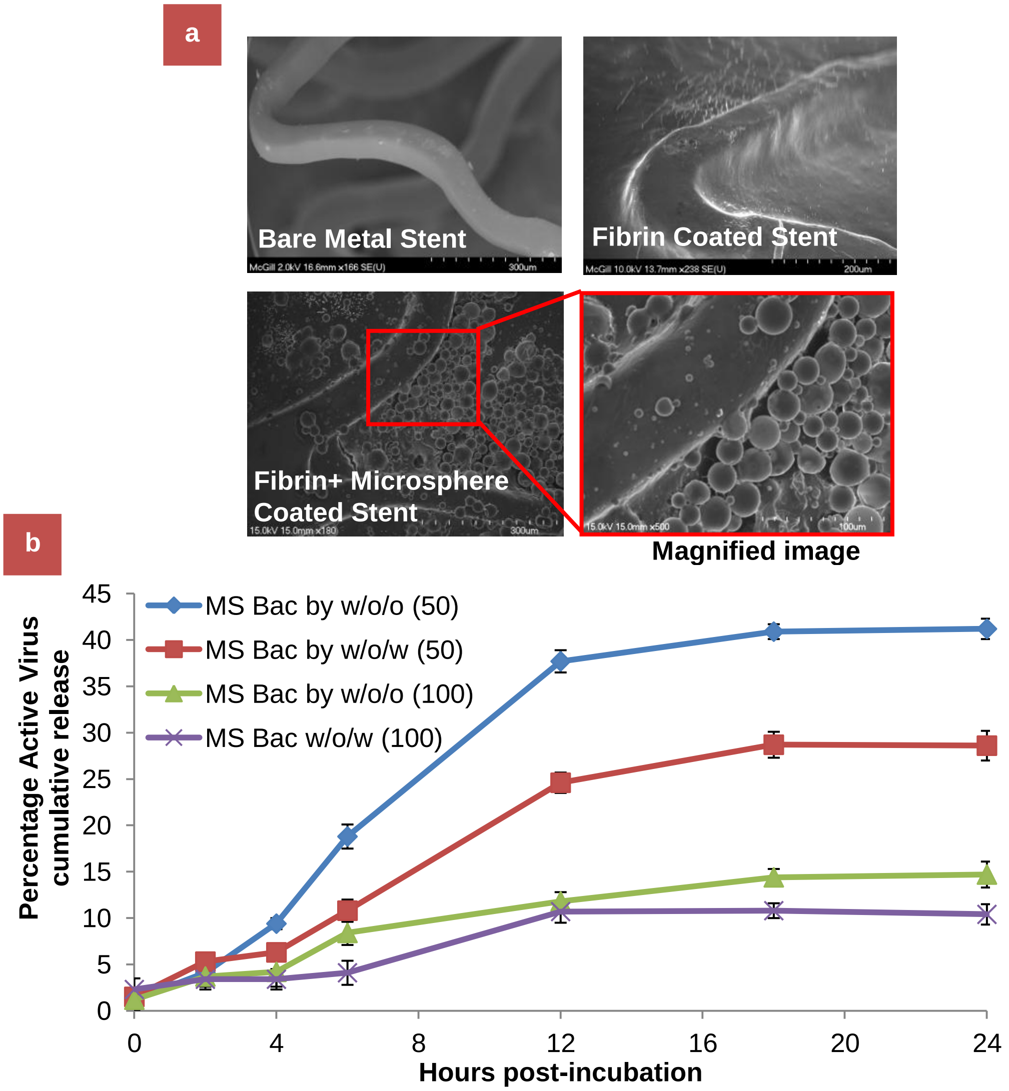

Figure S4: ***In vitro*** **bioctive stent characterization and release kinetics of nanaohybrids.**(a) SEM microphotographs of bare metal, fibrin coated and PLGA MS loaded fibrin coated stents. (b) Cumulative baculovirus release (in terms of % of initial virus loaded) from stents coated with varying concentrations (50 and 100 mg/ ml DCM) of PLGA MS prepared by w/o/w and w/o/o method shows controlled release of virus load over 24 h as a function of the PLGA concentration and MS preparation method.

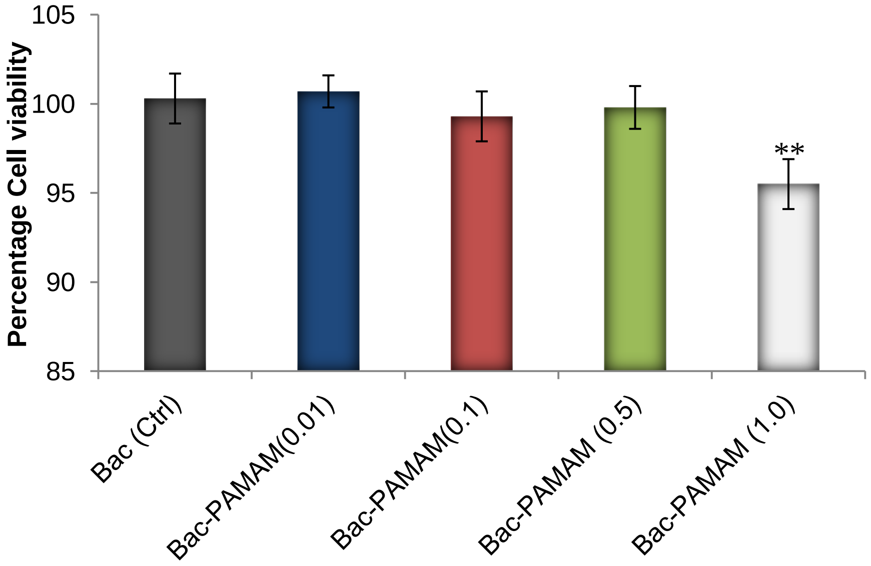

Figure S5: Cytotoxic effect of the the developed bioactive stent. The toxic effects of the different stent formulations on on HASMCs after 12h of incubation was analyzed. Data from different groups were represented in terms of percentage of viable cells. **=P<0.01 compared to control Bac group (n=3).

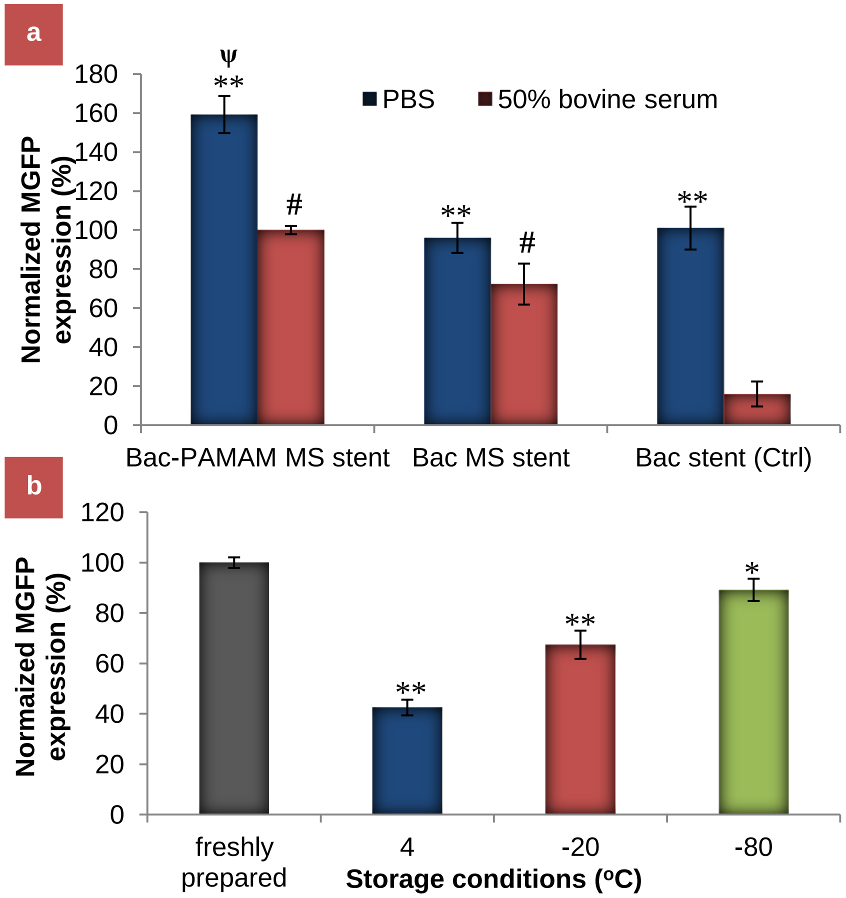

Figure S6: Effect of (a) serum and (b) storage temperatures on bioactivity of Bac_{MGFP} loaded stent. The Bac_{MGFP}-PAMAM MS, Bac_{MGFP} MS and Bac_{MGFP} stents, after 1 h incubation in 50% serum or PBS solution, were used to transduce 2x10^4 HASMCs per well in 96 well plate using standard method as mentioned in method section. Similarly, Bac_{MGFP}-PAMAM MS stents after storage for 3 months at different temperatures (4°C, -20°C, -80°C and control freshly prepared) were used to transduce HASMCs *in vitro*. Data was represented in terms of normalized MGFP expression taking Bac_{MGFP}-PAMAM MS expression as 100% (a) and taking freshly prepared stent expression as 100% (b). Data represent mean ± SD (n=3). Statistically significant differences within groups are denoted by ***=P<0.001 and **=P<0.01, while condition-matched differences between groups are represented by ψ<0.001 (PBS) and #<0.001 (serum).

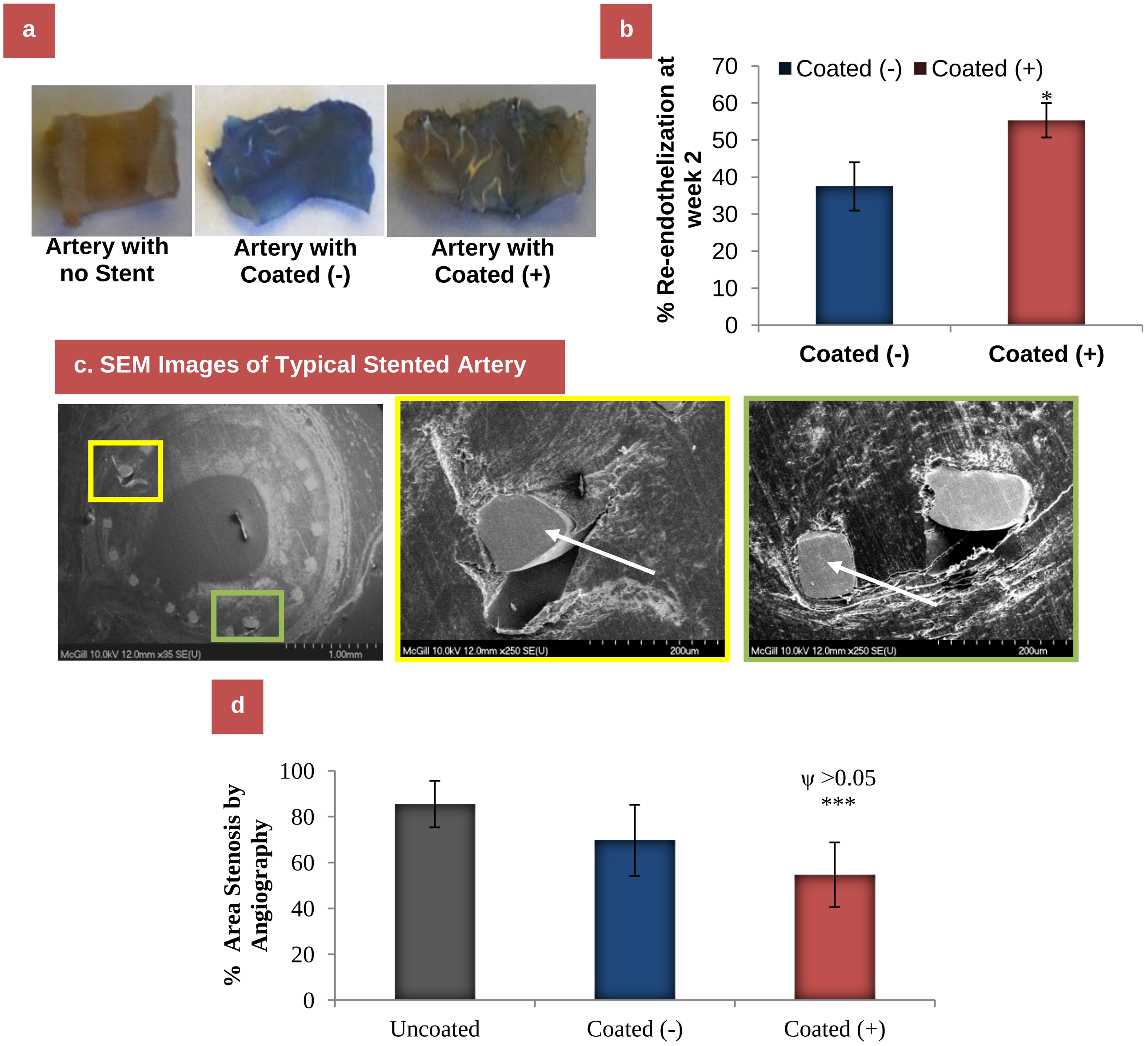

Figure S7: Re-endothelialization of vessels following stent implantation. (a). Evans blue staining confirms that Coated (+) group was able to recover the injury by endothelialization while in Coated (-) group the wounds was still exposed with high amount of Evans blue uptake. Control artery segment with no injury showed no signs of dye uptake. (b) Quantification of percentage re-endothelialization week 2 after staining using imaging software in the Coated (+) and Coated (-) group. The data represent the mean ±SD (n=3). (c) Cross-sectional view of stented artery through SEM demonstrating the intimal hyperplasia over the protruded stent struts. (d) Graphical representation of Angiographic image analysis data of the stented arteries demonstrate significantly reduced ISR in Coated (+) group compared to Uncoated group, although significance was not achieved when compared to Coated (-). The data represent the mean ±SD (n=8). ANOVA: ***=P<0.001. P value on comparing Coated (+) and Coated (-) is denoted by ψ.
